# Characterization of Aroma-Active Compounds in Seed Extract of Black Cumin (*Nigella sativa* L.) by Aroma Extract Dilution Analysis

**DOI:** 10.3390/foods7070098

**Published:** 2018-06-27

**Authors:** Songul Kesen, Armin Amanpour, Salwa Tsouli Sarhir, Onur Sevindik, Gamze Guclu, Hasim Kelebek, Serkan Selli

**Affiliations:** 1 Department of Food Processing, Naci Topcuoglu Vocational High School, Gaziantep University, 27600 Gaziantep, Turkey; 2 Department of Food Engineering, Faculty of Agriculture, Cukurova University, 01330 Adana, Turkey; dr.aamanpour@gmail.com (A.A.); foodengonursevindik@gmail.com (O.S.); gguclu@cu.edu.tr (G.G.); sselli@cu.edu.tr (S.S.); 3 Agri-Food Laboratory and Food Safety, Faculty of Sciences Dhar El Mahraz University Sidi Mohamed Ben Abdellah, B.P. 1796 Atlas, Fez, Morocco; salwa-sarhir@hotmail.fr; 4 Department of Food Engineering, Faculty of Engineering, Adana Science and Technology University, 01250 Adana, Turkey; hkelebek@adanabtu.edu.tr

**Keywords:** *Nigella sativa* L., black cumin seed, purge and trap extraction, volatile compounds, key odorants, GC-MS-Olfactometry

## Abstract

Turkish *Nigella sativa* L. seed extracts were used to detect the aroma and key odorant compounds of the spice using gas chromatography-mass spectrometry-olfactometry (GC-MS-O). Volatile compounds were extracted by the purge and trap extraction (PTE) method. A total of 32 volatile compounds consisting of different chemical classes acids (13), alcohols (7), phenols (3), terpene (1), esters (2), ketones (2), aldehyde (1), lactone (1) and hydrocarbons (2) were determined. The amounts of volatile compounds were found to be 21,544 µg kg^−1^. The application of aroma extract dilution analysis (AEDA) revealed the presence of 13 odor-active compounds alcohols (2), carboxylic acids (4), phenols (2), terpene (1), ketone (1), hydrocarbon (1) and unknown compounds (2) in *Nigella sativa* L. extract. Flavor dilution (FD) factors of key odorants ranged between 4 and 1024, while odor activity values (OAV) were in the range of 1.0 to 170.8. Acetoin was the only aroma-active ketone detected in *Nigella sativa* L. seed extracts. It had the strongest aroma (FD = 1024) and provided a buttery odor. This compound represented the most abundant compound of overall aroma profile with a concentration of 9394 µg kg^−1^, followed by isobutanoic acid (FD = 512 with a concentration of 218 µg kg^−1^) and contributed a powerful aroma and a cheesy characteristic odor.

## 1. Introduction

The annual flowering plant of *Nigella sativa* (*N. sativa*), commonly known as black seed and black cumin, has been used for public medicine in regions like India, Africa and Asia. The seeds and oils of black cumin are used in herbal medicine all over the world for the treatment and prevention of a number of diseases and conditions that include asthma, diarrhea and dyslipidaemia [1].

Black cumin seed (*N. sativa* L.) gives a pungent aroma when added to food and is especially used in bread and bakery products in Turkey and Arabic countries. The black cumin seed oil, which is usually extracted using the cold pressing system, has an important place in cosmetics, cooking, and particularly in pharmacy. In addition, black cumin seed oil has stronger oxidative stability compared to other vegetable oils due to sterols, tocopherols, and phenolic compounds [2,3]. Black cumin seed extract is also traditionally used in many cancer treatments in Turkey and other countries.

Black cumin seed oil consumption could have an important role in the health and nutrition of humans due to its special fatty acid composition. In addition, it contains fat-soluble bioactives that are usually found in precious oil, such as tocols and sterols [2,4]. *N. sativa* L. has a lot of healing properties, such as improving inflammatory, diabetes, cardiovascular, renal, and liver diseases. Chemical compounds of black cumin include fixed oils (35–40%), volatile compounds (0.05%), proteins (23%), various amino acids, sugars, mucilages, alkaloids, organic acids, tannins, resins, lipases, phytosterols, vitamins, and various types of minerals as well as timolol thymoquinone and di-thymico-quinone which are derived from ferrous oil of *Nigella sativa* L. [5].

Aroma compounds are one of the most important agents as they shape the quality of food and affect consumer preferences. Among many chemical compounds, aroma is the main agent that clarifies the organoleptic properties of foods. Only a small part of the huge number of aroma substances actually contributes to the global aroma. Aroma-active compounds can be detected in the complicated structure of hundreds of aroma compounds with the aid of an olfactometric technique. The gas chromatography-olfactometry (GC-O) technology has made it feasible to classify volatile compounds into aroma-active and non-aroma-active compounds with respect to their concentration in the samples examined [6,7].

Some properties of black cumin seeds and their oils have been investigated by different researchers. Kiralan et al. investigated the physicochemical properties and stability of the seed oil of *Nigella sativa* L., which changed with various extraction methods [8]. Changes in volatile compounds by microwave heating were also studied in black cumin oil [3]. In another study, volatile compounds in the seeds of *Nigella sativa* L. from two different regions (Bangladesh and Egypt) were characterized by Liu et al. [9].

To the best of our knowledge, no study has so far been reported to determine the key odorants of *N. sativa* L. using gas chromatography-mass spectrometry-olfactometry (GC-MS-O). Consequently, the present study had three aims: (1) identify aroma compounds of *Nigella sativa* L. extracts by purge and trap extraction (PTE) system; (2) describe aroma-active compounds of Turkish *Nigella sativa* L. extracts using aroma extract dilution analysis (AEDA) with the aid of GC-MS-O; and (3) determine sensory properties of black seed extract.

## 2. Materials and Methods

### 2.1. Chemicals

The water used in the analysis was cleansed by a Millipore-Q system (Millipore Corp., Saint-Quentin, France). Anhydrous sodium sulfate, dichloromethane, and 4-nonanol (IS) were purchased from Merck (Darmstadt, Germany). Standard aroma compounds used in the study were supplied from Sigma Aldrich (Sigma-Aldrich, Steinheim, Germany).

### 2.2. N. sativa (Black Cumin) Seed Extract

*Nigella sativa* seed extract was purchased from a herbalist in Bodrum, Turkey in July 2017. The seed extracts were stored in a brown glass bottle and maintained at 4 °C until aroma analysis.

### 2.3. Extraction of Volatile Compounds

The volatile compounds of seed extract were isolated by aid of the purge and trap technique. This method is applied in special cartridges (C18) with the help of nitrogen carrier gas controlled by a flowmeter. The flowmeter is linked to a separator system to split the flow in several passages in order to purge samples therewithal. The needles of the cartridge and nitrogen gas source were inserted to septum of vial to purge and trap the volatile compounds. Lichrolut EN resin (200 mg, Merck, Darmstadt, Germany) was used as adsorbent, which was identified previously as the optimal material for retention of aroma compounds [10]. Briefly, the 10 mL of sample was sited in a 20 mL bottle from which oxygen was removed from the head space using nitrogen gas. The samples were exposed to preincubation at the temperature of 60 °C for 10 min. Then, extraction treatment was completed for 90 min with a 500 mL min^−1^ of nitrogen flow. After that, the compounds trapped in the cartridge were removed slowly and vigorously eluted with 12 mL of dichloromethane. The mixture of volatile compounds and dichloromethane was dehydrated by anhydrous sodium sulfate, and the aromatic extract was then concentrated to 5 mL using Kuderna Danish concentrator (Sigma Aldrich, St. Louis, MO, USA). Finally, it was condensed to 200 μL under nitrogen flow. The aromatic extracts were preserved at −20 °C in a 2 mL glass vial until analysis. Each procedure was repeated in triplicate, and the concentration of volatiles was expressed by 4-nonanol with ethyl alcohol as internal standard (with the concentration of 43.27 mg L^−1^) equivalent.

### 2.4. GC/FID and GC/MS/O Analysis of Aroma Compounds

A GC (Agilent 6890, Santa Clara, CA, USA) equipped with a flame ionization detector (FID), a mass selective detector (Agilent 5973 MSD) and a Gerstel ODP 2 (Linthicum, MD, USA) sniffing port using a deactivated capillary column (30 cm × 0.3 mm) heated at 240 °C and supplied with humidified air at 40 °C was used to analyze aroma and aroma-active compounds. Helium was used as a carrier gas with a flow rate of 1.5 mL min^−1^. Aroma compounds were separated on DB-Wax column (30 m length × 0.25 mm i.d. × 0.5 μm thickness, Santa Clara, CA, USA). The program conditions were as follows: The oven start temperature was 50 °C (1 min), the subsequent gradient was 5 °C min^−1^ to 200 °C and then at a rate of 8 °C min^−1^ to 260 °C with a final hold at 260 °C for 5 min (DB-WAX column, Santa Clara, CA, USA). Afterwards, GC effluent was split 1:1:1 among the FID, MSD, and sniffing mode via a Dean’s switch. A total of 3 μL of extract was injected each time in pulsed splitless (40 psi; 0.5 min) mode. Mass spectra in the electron ionization mode were recorded at 70 eV and a mass/charge range of 30–300 amu at 2.0 scan s^−1^ scan rate. The identification of compounds was carried out using mass spectral database (NIST 98, Wiley 6), retention index, and chemical standards. Concentration of each compound was calculated using internal standard method [11].

### 2.5. Aroma Extract Dilution Analysis 

To characterize the key aroma compounds, an AEDA was used. For this analysis, the condensed aroma extracts were diluted one by one in a rate of 1:1, 1:2, 1:4, 1:8, 1:16, …, 1:1024, etc. using dichloromethane. The individual aroma extracts were smelled by three experienced panelists in olfactometric port of GC/MS/O [12]. Sniffing of diluted extracts was sustained until there was no odor sense. Each perceived odor was expressed as flavor dilution (FD) factor, such as 2, 4, 8, …, 1024, etc., corresponding to the above ratios. The higher the FD factor value of aroma-active compound, the more effect on the aroma profile [13,14].

### 2.6. Sensory Descriptive Analysis

#### 2.6.1. Panel

The panel consisted of ten assessors (four females and six males between 24 and 46 years of age) from the Biotechnology Laboratory at the Food Engineering Department of Cukurova University. The panelists were coached in scent distinction and sensorial assessment methods and had experience in GC-MS analysis.

#### 2.6.2. Preparation and Presentation of the Samples

There are various ways in which the representativeness of the aroma extracts of such studies can be estimated. In this work, a cardboard sniffing strip (Granger-Veyron, Lyas, France) was used to investigate the properties of the aroma extracts provided by the purge and trap extraction method. In previous studies, cardboard strips have been used for the representativeness tests of olive oil [15], dill, and savory spice [16] extracts. As a reference, 5 mL of black cumin seed extract was placed in a brown coded flask (25 mL) for representativeness tests. The aroma extracts of samples obtained using the purge and trap technique were adsorbed on the cardboard. The details of the work have been given in our previous study [17].

#### 2.6.3. Intensity and Similarity Tests

These tests were applied to discover the relationship between the black cumin seed extract and its aromatic extract. The panelists were told to smell and compare the aroma of the reference sample and aroma extract to determine the similarity and intensity of their scents. For each test, a 10 cm scale was used. If the odor was very different from the reference, it was scored left-based; if the odor was alike to the reference, it was marked right-based. The distance marked on the scale was read as centimeters from the left.

#### 2.6.4. Descriptive Analysis

Nine descriptors buttery, cheesy, citrusy, balsamic, smoky, putrid fruit, burnt plastic, fatty, and green that define their decisive aroma were assigned by the expert and trained panelists. The reference sample and aromatic extract were offered to the panelists who were requested to characterize the odor properties of samples by judging the consistency of the above-mentioned descriptor on a scale of 10 cm. If there was no odor it was scored at the left end, while a very strong odor at the right end. The scores given by each panelist were determined on the scale and then the average value of the odor intensity was calculated as centimeters.

#### 2.6.5. Statistical Analysis

The independent-samples analysis of variance test (*t*-test), which compares the sensorial scores of the original sample and their extracts, was used as the statistical method. All data obtained in analysis were given as averages of ten replicates. SPSS Statistics software version 22.0 (Chicago, IL, USA) was used for statistical analysis.

## 3. Results and Discussion

### 3.1. Sensory Analysis

#### 3.1.1. Odor Sensory Profiles

**Figure 1:**
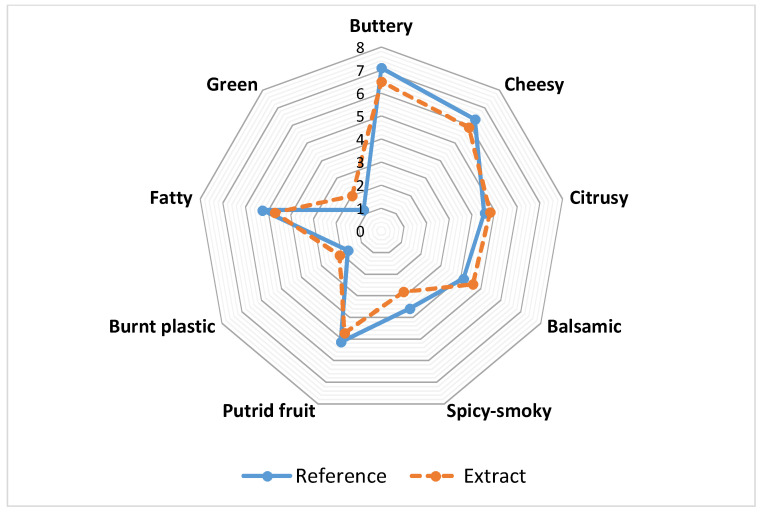
Odor sensory features of black cumin seed extract and its aromatic extract.

Ten panelists compared the aromatic extracts from black cumin seeds to reference samples (seed extract of black cumin) for odor. A spider graph of the reference sample and its aromatic extracts was created with nine descriptors determined by the panelists (Figure 1). As shown in Figure 1, odor descriptors of a reference sample and its extracts were described as buttery, cheesy, citrusy, balsamic, spicy-smoky, putrid fruit, burnt plastic, fatty, and green. Among the descriptors, buttery and cheesy notes were the most intense and received the highest scores, while citrusy, balsamic, spicy-smoky, putrid fruit, burnt plastic, fatty, and green represented the lower scores. From Figure 1 it is obvious that panelists evaluated the sensory properties of aromatic extract similar to the original sample. In addition, there were no statistical differences (*p* < 0.05) between the reference sample and aromatic extract for the nine descriptors. According to the results which were consistent with the sensory analysis, it was shown that the most dominant aroma-active compound was acetoin. It provided a buttery scent that is known to be formed via enzymatic activity. This result suggests that the buttery and cheesy scents caused by aroma-active compounds play a vital role in black cumin’s unique flavor.

#### 3.1.2. Similarity and Intensity Evaluation of Aromatic Extract

According to the results, both the similarity and intensity scores were satisfactory. The similarity score of the aromatic extract obtained from the PTE method on smelling strips was found to be 7.9 cm on a 10 cm scale. The mean intensity score obtained from the same extract was 7.1 cm. When compared with the extant literature [15,17], the similarity and intensity scores of the extract was determined to be at a suitable level for olfactometric analysis.

### 3.2. Aroma Compounds of Black Cumin Seed Extract

**Figure 2:**
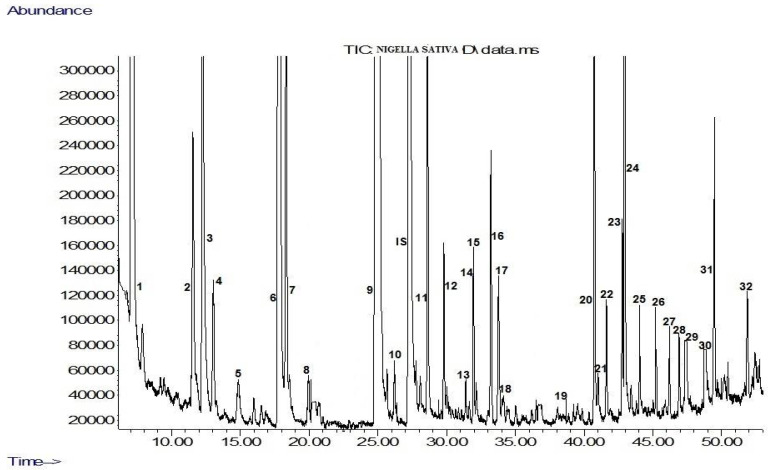
Gas chromatography-mass spectrometry (GC-MS) chromatogram of black cumin seed extract. 1: 2-Methyl-3-butanol, 2: *m*-xylene, 3: 3-penten-2-ol, 4: limonene, 5: styrene, 6: acetoin, 7: hydroxyacetone, 8: (*Z*)-2-methyl-2-buten-1-ol, 9: acetic acid, 10: furfural, IS: internal standard (4-nonanol), 11: propanoic acid, 12: isobutanoic acid, 13: butyrolactone, 14: butanoic acid, 15: propenoic acid, 16: furfuryl alcool, 17: pentanoic acid, 18: benzyl acetate, 19: 2-(2-ethoxyethoxy) ethanol, 20: hexanoic acid, 21: guaiacol, 22: benzyl alcohol, 23: phenethyl alcohol, 24: (*E*)-3-hexenoic acid, 25: heptanoic acid, 26: phenol, 27: octanoic acid, 28: eugenol, 29: nonanoic acid, 30: ethyl 4-ethoxybenzoate, 31: hexadecanoic acid, 32: octadecanoic acid.

The volatile compounds detected in black cumin seed extract are given in Table 1 and its GC-MS chromatogram is shown in Figure 2. In Table 1, mean values (µg kg^−1^) of the GC analyses of triplicate extractions and standard deviations are reported. As can be seen in Table 1, a wide variety of volatile compounds was found in the black cumin seed extract. A total of 32 volatile compounds consisting of different chemical classes were determined. These compounds were acids (13), alcohols (7), phenols (3), terpene (1), esters (2), ketones (2), aldehyde (1), lactone (1), and hydrocarbons (2). The amount of volatile compounds was 21,544 µg kg^−1^. The most abundant volatile compound was acetoin (9394 µg kg^−1^), followed by acetic acid (3088 µg kg^−1^).

Ketones were quantitatively and qualitatively the most dominant volatiles in the extracts (10,202 µg kg^−1^). The highest amount of ketone compounds found was acetoin (9394 µg kg^−1^). It is a pale to yellowish liquid with a delightful yogurt creamy odor and a fatty butter taste. It is a volatile compound broadly used in foods, detergents, cosmetics, cigarettes, chemical synthesis, biological pest controls, and plant growth promoters. It works mainly as flavor and fragrance [18]. In another study related with yoghurt, acetoin was denoted as one of the compounds contributing most to the typical aroma and flavor [19]. Buttery et al. found that acetoin (3-hydroxy-2-butanone) was one of the main volatile compounds in rice cakes [20].

Carboxylic acids were the second largest class of aroma compounds, with 6574 µg kg^−1^. Among the carboxylic acids detected by GC-MS, acetic acid was identified as having the highest amount (3088 µg kg^−1^). This compound was successfully identified and quantified in olive oil [10] and dry salted black table olive [21] in our previous studies by using purge and trap extraction technique. It has been stated by Sabatini et al. that acetobacter causes acetic acid formation by ethanol oxidation [22]. Pico et al. found that acetic acid is one of the main volatile compounds formed during fermentation in dough [23].

Alcohols were the third largest class of aroma compounds. A total of seven alcohols were found in the samples. The total amount of volatile alcohols was 3621 µg kg^−1^. Among the volatile alcohols, 2-methyl-3-butanol was the most abundant alcohol compound, with 2627 µg kg^−1^. The action of alcohol dehydrogenase (ADH) enzyme produces alcohols. It is commonly found in plants and is in charge of the formation of volatile alcohols that deliver the aroma of foods.

Two hydrocarbon compounds (422 µg kg^−1^) m-xylene and styrene were identified and quantified in the extract. M-xylene was the most abundant among these compounds (357 µg kg^−1^).

Terpene (176 µg kg^−1^), phenols (226 µg kg^−1^), and aldehydes (144 µg kg^−1^) were found in low quantities. Esters (131 µg kg^−1^) and lactone (48 µg kg^−1^) compounds were also detected in low quantities. Esters are responsible for the fruity notes. Increasing the alcohol acetyl transferases (AAT) activity can enhance the production of volatile esters in foods [11]. Lactones contribute to the characteristic fruity note [24].

### 3.3. GC-MS-O Results

To determine the aroma-active compounds, the representative aromatic extract obtained by PTE system from the black cumin seed extract was analyzed by GC-MS-O. Most of the aroma-active compounds were identified for the first time in *Nigella sativa* L. extract which revealed a total of 13 odor-active compounds alcohols (2), carboxylic acids (4), ketone (1), phenols (2), hydrocarbon (1) and terpene (1 with FD factor ranging between 4 and 1024, while the odor activity values (OAV) ranged between 1.0 and 170.8 (Table 2). Two unknown compounds with FD values ≤ 32 perceived by GC-MS-O, but not identified by GC-MS, were also detected (Table 2).

The characteristic odor of black cumin extract is dominated by buttery, cheesy, balsamic, citrusy, fatty, and spicy-smoky odors consistent with the description notes of sensory analyses results of aromatic extract obtained by PTE method. Additionally, putrid fruit, burnt plastic, honey/floral, and green odor descriptors were relatively attributed to lower FD factors.

Acetoin was the only aroma-active ketone detected in black cumin extract. It had the strongest aroma (FD = 1024), providing a buttery odor. This compound represented the most abundant compound of overall aroma profile with a concentration of 9394 µg kg^−1^, followed by isobutanoic acid (FD = 512), which had a powerful aroma of cheesy characteristic odor. Despite the highest concentration of acetic acid (3088 µg kg^−1^), its contribution to the total scent of black cumin extract was absent in the case of olfactometric and sensory analysis.

Limonene (terpene) and styrene (hydrocarbon) contributed to the overall aroma representing the same flavor dilution factor of 256, which associated with citrusy and balsamic odor notes, respectively.

The phenol groups are represented in black cumin extract by two important key aroma compounds eugenol (FD = 128) and guaiacol (FD = 64) providing a high FD factor with a typical odor of spicy and smoky notes.

Carboxylic acid group was another important aroma-active compound found in black cumin extract. Along with isobutanoic acid (FD = 512, cheesy odor), propanoic acid (FD = 128, fatty odor), hexanoic acid (FD = 64, cheesy odor) and pentanoic acid (FD = 32, cheesy odor) were also detected by olfactometry analysis. Therefore, typical odor properties of black cumin extract showed dairy product odorant attributes.

Phenethyl alcohol (FD = 16, honey/floral) and 3-penten-2-ol (FD = 4, green odor) were detected as aroma-active alcohols, with a negligent effect on odor of *Nigella sativa* L. extract. The contribution of these two compounds was undetectable or at trace levels in the sensory analysis because of their high odor threshold values.

Among the overall key aromas of *Nigella sativa* L. extract, two unknown compounds existed with a considerable flavor dilution factors to a characteristic odor of this sample. Unknown 1 (LRI = 1158, FD = 16) was a potent aroma providing a burnt plastic odor, while unknown 2 (LRI = 1394) provided putrid fruit aroma with a FD factor of 32.

## 4. Conclusions

To the best of our knowledge, this is the first report to date assessing aroma-active compounds in seed extracts of Turkish black cumin. Ketone and acid compounds were the major chemical group among the detected aroma compounds, followed by alcohols. Among the volatile compounds, acetoin was the most abundant volatile compound, followed by acetic acid. According to data obtained from olfactometric analysis, characteristic odor in black cumin seed extracts were dominated by buttery, cheesy, balsamic, citrusy, fatty, and spicy odors. This is consistent with the description notes of sensory analyses of seed (sample) and their aromatic extracts. Additionally, putrid fruit, burnt plastic, honey/floral, and green odor descriptors were relatively attributed to lower FD factors.

## Figures and Tables

**Table 1:** Volatile compounds of black cumin seed extract.

No.	LRI ^a^	Compounds	Concentration ^b^	Identification ^c^
		Carboxylic acids		
1	1450	Acetic acid	3088 ± 28.2	LRI, MS, Std
2	1538	Propanoic acid	1187 ± 40.8	LRI, MS, Std
3	1562	Isobutanoic acid	218 ± 2.0	LRI, MS, Std
4	1628	Butanoic acid	138 ± 4.6	LRI, MS, Std
5	1638	Propenoic acid	147 ± 6.6	LRI, MS, tent
6	1731	Pentanoic acid	176 ± 4.5	LRI, MS, Std
7	1854	Hexanoic acid	309 ± 6.1	LRI, MS, Std
8	1954	(*E*)-3-Hexenoic acid	516 ± 13.8	LRI, MS, Std
9	1960	Heptanoic acid	126 ± 4.1	LRI, MS, Std
10	2047	Octanoic acid	112 ± 3.8	LRI, MS, Std
11	2178	Nonanoic acid	95 ± 4.5	LRI, MS, Std
12	2930	Hexadecanoic acid	346 ± 2.5	LRI, MS, Std
13	3181	Octadecanoic acid	116 ± 2.1	LRI, MS, Std
		Total	6574	
		Alcohols		
14	1121	2-Methyl-3-butanol	2627 ± 7.8	LRI, MS, Std
15	1182	3-Penten-2-ol	394 ± 2.7	LRI, MS, Std
16	1332	(Z)-2-Methyl-2-buten-1-ol	110 ± 2.2	LRI, MS, Std
17	1651	Furfuryl alcohol	227 ± 4.7	LRI, MS, Std
18	1796	2-(2-Ethoxyethoxy) ethanol	9 ± 0.1	LRI, MS, tent
19	1881	Benzyl alcohol	123 ± 1.9	LRI, MS, Std
20	1916	Phenethyl alcohol	131 ± 0.0	LRI, MS, Std
		Total	3621	
		Phenols		
21	1873	Guaiacol	12 ± 0.1	LRI, MS, Std
22	1989	Phenol	125 ± 1.8	LRI, MS, Std
23	2158	Eugenol	89 ± 1.1	LRI, MS, Std
		Total	226	
		Terpene		
30	1205	Limonene	176 ± 0.3	LRI, MS, Std
		Total	176	
		Esters		
24	1740	Benzyl acetate	46 ± 3.6	LRI, MS, Std
25	2593	Ethyl 4-ethoxybenzoate	85 ± 2.0	LRI, MS, tent
		Total	131	
		Ketones		
26	1320	Acetoin	9394 ± 87	LRI, MS, Std
27	1326	Hydroxyacetone	808 ± 24.7	LRI, MS, Std
		Total	10,202	
		Aldehyde		
28	1470	Furfural	144 ± 3.1	LRI, MS, Std
		Total	144	
		Lactone		
29	1614	Butyrolactone	48 ± 0.5	LRI, MS, Std
		Total	48	
		Hydrocarbons		
31	1132	M-xylene	357 ± 17.6	LRI, MS, tent
32	1270	Styrene	65 ± 1.4	LRI, MS, tent
		Total	422	
		General Total	21,544	

^a^ LRI: retention indices on DB-WAX column. ^b^ Concentration: Mean values based on three repetitions as µg kg^−1^. ^c^ Identification: Methods of identification; LRI (linear retention index), MS tent (tentatively identified by MS), Std (chemical standard). When only MS or LRI is available for the identification of compounds, it must be considered as an attempt of identification.

**Table 2:** Aroma-active compounds of black cumin seed extract (FD ≥ 4).

No.	Aroma-Active Compounds	LRI ^a^	Odor Descriptions ^b^	FD ^c^	OT (ppm) ^d^	OAV ^e^
1	Unknown I	1158	Burnt plastic	16	-	-
2	3-Penten-2-ol	1182	Green	4	0.4 [25]	1.0
3	Limonene	1205	Citrusy	256	0.01 [26]	17.6
4	Styrene	1270	Balsamic	256	0.0036 [27]	18.2
5	Acetoin	1320	Buttery	1024	0.055 [28]	170.8
6	Unknown II	1394	Putrid fruit	32	-	-
7	Propanoic acid	1538	Fatty	128	0.1 [29]	11.9
8	Isobutanoic acid	1562	Cheesy	512	0.01 [29]	22.0
9	Pentanoic acid	1731	Cheesy	32	0.07 [30]	2.5
10	Hexanoic acid	1854	Cheesy	64	0.093 [31]	3.3
11	Guaiacol	1873	Smoky	64	0.003 [32]	3.8
12	Phenethyl alcohol	1916	Honey, floral	16	0.086 [28]	1.5
13	Eugenol	2158	Spicy-smoky	128	0.006 [30]	14.8

^a^ Linear retention index calculated on DB-WAX capillary column. ^b^ Odor description as perceived by panelists during olfactometry. ^c^ Flavor dilution (FD) factor is the highest dilution of the extract at which an odorant was detected by aroma extract dilution analysis. ^d^ Odor thresholds in water. These values were obtained from the following References [25,26,27,28,29,30,31,32]. ^e^ The odor activity values (OAV) were obtained by dividing concentration of the compounds by their threshold.
